# Clinical efficacy and mechanistic evaluation of aflibercept for proliferative diabetic retinopathy (acronym CLARITY): a multicentre phase IIb randomised active-controlled clinical trial

**DOI:** 10.1136/bmjopen-2015-008405

**Published:** 2015-09-14

**Authors:** Sobha Sivaprasad, A Toby Prevost, James Bainbridge, Rhiannon Tudor Edwards, David Hopkins, Joanna Kelly, Phil Luthert, Caroline Murphy, Jayashree Ramu, Negin Sarafraz-Shekary, Joana Vasconcelos, Beverley White-Alao, Philip Hykin

**Affiliations:** 1 NIHR Moorfields Biomedical Research Centre, London, UK; 2 KCL Department of Primary Care and Public Health Sciences, NIHR Biomedical Research Centre at Guy's and St Thomas’ NHS Foundation Trust, and King's College London, London, UK; 3 NIHR Moorfields Biomedical Research Centre, London, Institute of Ophthalmology, London, UK; 4 Centre for Health Economics and Medicines Evaluation, Bangor University, Bangor, Gwynedd, UK; 5 Department of Diabetes and Endocrinology, King's College Hospital NHS Foundation Trust, London, UK; 6 King's Clinical Trials Unit at KHP, Kings College London, Institute of Psychiatry, London, UK; 7 Institute of Ophthalmology, London, UK

## Abstract

**Introduction:**

Proliferative diabetic retinopathy (PDR) is the main cause of severe visual loss in people with diabetes mellitus. The standard treatment for this condition is panretinal photocoagulation (PRP). This laser treatment is inherently destructive, with predictable adverse effects on visual function, and a safer alternative is required. Intravitreal injection of vascular endothelial growth factor (VEGF) inhibitors can induce short-term regression of retinal neovascularisation. The aim of this randomised controlled trial is to determine the efficacy, safety and cost-effectiveness of intravitreal aflibercept, an inhibitor of VEGF-A, VEGF-B and placental growth factor (PLGF), in PDR, and to investigate the impact on local oxygenation.

**Methods and analysis:**

This is a phase IIb randomised controlled single-masked multicentre clinical trial to determine the impact of repeated intravitreal aflibercept injections in the treatment and prevention of PDR. 220 participants with treatment-naïve or treated but active retinal neovascularisation in at least one eye will be randomly allocated 1:1 to intravitreal aflibercept injections or PRP for a period of 52 weeks. The primary outcome is the change in best-corrected visual acuity in the study eye at 52 weeks. Secondary outcomes include changes from baseline in other visual functions, anatomical changes and cost-effectiveness. Ocular and non-ocular adverse events will also be reported over 52 weeks.

**Ethics and dissemination:**

The study has been approved by the National Research Ethics Service (NRES) committee with respect to scientific content and compliance with applicable research and human subjects’ regulations. Findings will be reported through scientific publications and research conferences. The results of this study will provide clinical evidence for the feasibility, efficacy safety and cost-effectiveness of intravitreal aflibercept for PDR.

**Trial registration number:**

ISRCTN 32207582.

## Background

Diabetic retinopathy (DR) is the most common complication of diabetes and is caused by progressive damage to the retinal blood vessels with increasing duration of diabetes.^1^ The two major sight-threatening complications of DR are diabetic macular oedema (DMO) and proliferative diabetic retinopathy (PDR).^2^ ^3^ PDR is characterised by growth of new blood vessels that can cause severe sight loss as a result of vitreous haemorrhage, retinal detachment and neovascular glaucoma (NVG).

Multiple molecular mechanisms are involved in the pathogenesis of DR. However, a final common pathway involves retinal hypoxia and consequent upregulation of vascular endothelial growth factor (VEGF).^4^ Therefore, treatment options for PDR aim either to promote retinal oxygen availability or to inhibit VEGF. Panretinal photocoagulation (PRP) is applied to the peripheral retinal tissue to ablate areas of the peripheral retina and thereby reduce retinal oxygen consumption.^5^ Increased oxygen availability in an unlasered retina downregulates VEGF production, inducing regression of retinal neovascularisation (NV). However, PRP-induced regression of new vessels is variable, and although timely PRP can protect visual acuity, serious adverse effects are common.^3^ ^6^ The development or worsening of pre-existing macular oedema causes vision loss in 13%. In addition, loss of peripheral vision, night vision or contrast sensitivity affects nearly 5%. Non-responders and severe cases may also require vitrectomy. Nine-month follow-up of 209 eyes with PDR treated with PRP in the National Health Service (NHS) showed that 46% did not reach the driving standard, of whom 13% had a poor visual acuity outcome of ≤6/60 Snellen.^6^ An alternative treatment option that could either obviate or delay the need for PRP treatment for PDR would therefore be desirable.

Novel intravitreal anti-VEGF therapies including aflibercept, ranibizumab and bevacizumab have substantially improved the treatment prognosis for a wide range of ocular diseases, including neovascular age-related macular degeneration, DMO and retinal vein occlusions. Anti-VEGF treatment has superseded macular laser treatment and is now the standard of care for DMO involving the central macula *(*http://www.nice.org.uk*).* Several clinical and preclinical studies indicate that VEGF is a key mediator in the development of retinal NV. Injection of VEGF into the eye of a non-human primate stimulates growth and permeability of new vessels on the retina, simulating PDR, and also induces NVG.^7^ There is also clear evidence that hypoxic retina produces VEGF.^8^ Levels of VEGF mRNA and protein are elevated in a manner that is spatially and temporally consistent with the role for VEGF in the growth of new vessels.^9^ VEGF levels are highest in ocular fluid in patients with PDR compared with other retinal diseases.^10^ Evidence in support of a direct role of anti-VEGF agents blocking retinal new vessel growth has also been reported using a soluble VEGF receptor, anti-VEGF aptamers and VEGFR1-neutralising antisera.^11^ ^12^ Recent evidence also indicates that monthly anti-VEGF treatment can reduce the severity and delay the progression of DR over 24 months.^13^ Several case series using different anti-VEGF agents have shown that anti-VEGF therapy is effective in causing transient regression of retinal NV in PDR.^14^ The impact of this treatment on visual function and the effects of these agents on retinal NV compared with PRP remain unclear. It is possible that a long-acting anti-VEGF agent such as aflibercept may be sufficient to preclude the need for laser treatment as long as the eye continues to receive the treatment. Accordingly, we need to investigate this further by conducting a robust multicentre randomised controlled trial comparing the efficacy, safety and cost-effectiveness of repeated intravitreal aflibercept relative to PRP in treating and preventing the recurrence of PDR.

Currently, there are two multicentre trials evaluating the efficacy of ranibizumab in PDR (clinicaltrials.gov). However, these studies are including only participants previously untreated with high-risk PDR, a group that is less prevalent in the UK owing to the relatively prompt managment of early PDR identified by the nationwide screening programmes. Furthermore, these trials exclude cases that have been partially treated or are poorly responsive to prior laser therapy. The aim of the proposed study is to determine the impact of anti-VEGF therapy in a typical UK patient cohort with PDR. A mechanistic substudy will further explore the pathogenesis of PDR in terms of whether repeated intravitreal aflibercept retards the progression of PDR by (1) causing regression of retinal NV, (2) improving oxygen saturation within retinal vessels and (3) reducing quantifiable areas of retinal non-perfusion.

## Study design

**Figure 1:**
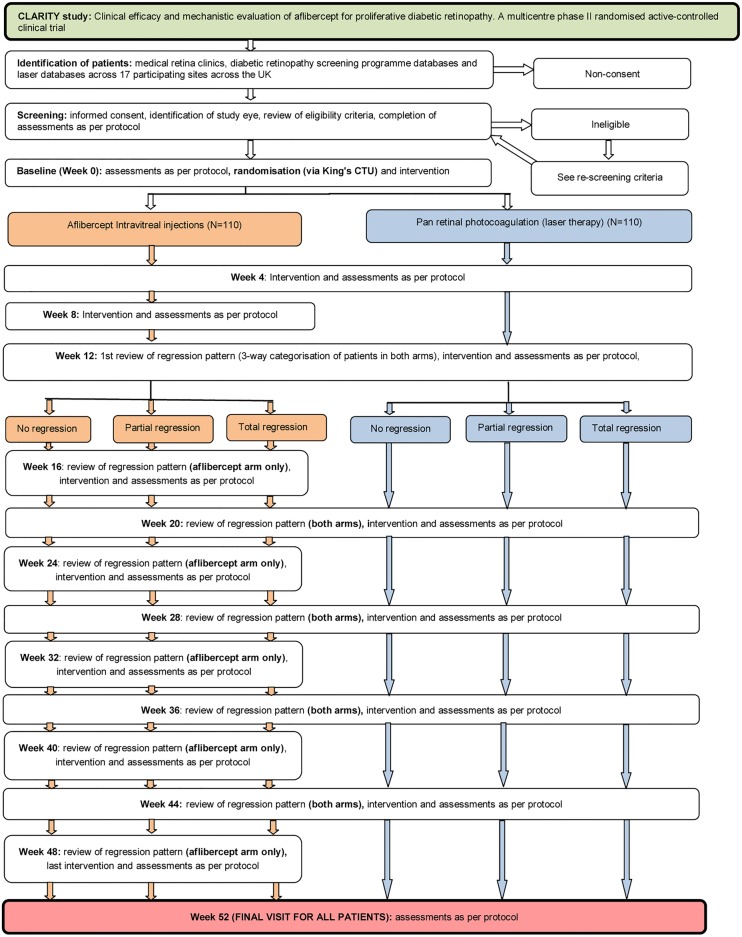
The trial design showing the assessments in each arm (CTU, Clinical Trials Unit).

This is a phase IIb randomised controlled single-masked multicentre clinical trial that will test the non-inferiority of intravitreal aflibercept to the standard of care of PRP on 220 participants with PDR at 52 weeks. Forty willing participants (20 in each arm) will also undergo retinal oximetry as part of the mechanistic substudy (see figure 1 for the trial flow diagram).

Inclusion criteria:

1. Participants of either sex aged 18 years or over.
2. Diagnosis of diabetes mellitus (type 1 or 2).
3. Best-corrected visual acuity (BCVA) in the study eye better than or equal to 54 Early Treatment Diabetic Retinopathy Study (ETDRS) letters (Snellen visual acuity 6/24).
4. Visual acuity in fellow eye ≥2/60.
5. PDR with no evidence of previous PRP or presence of new or persistent retinal NV despite prior PRP that (a) requires treatment in the opinion of the investigator and (b) there is sufficient space in the peripheral retina to perform more PRP treatment. In patients with both eye involvements, the eye with no PRP or the least number of PRP burns will be randomised as the study eye. If both eyes have had no previous PRP, the eye with the better visual acuity will be randomised as the study eye. However, patients will be offered a choice and can opt for the ‘worse seeing eye’ to be randomised.
6. Media clarity, pupillary dilation and participant cooperation sufficient for adequate fundus photographs. Eyes with mild pre-retinal haemorrhage or mild vitreous haemorrhage that does not interfere with clear visualisation of the macula and optic disc are eligible for this study.
7. Ability to give informed consent.
8. Women should use effective contraception, be post-menopausal for at least 12 months prior to trial entry, or surgically sterile.

Key exclusion criteria include coexistent ocular disease in the study eye that may interfere with visual outcome, treatment or trial assessments; moderate or dense vitreous haemorrhage that prevents clear visualisation of the macula and/or optic disc or prevents PRP treatment; significant fibrovascular proliferation or tractional retinal detachment in the posterior pole; prior vitrectomy; presence of centre-involving macular oedema at baseline; iris or angle NV and NVG; anticipated need for cataract extraction or vitrectomy within the next 12 months; previous intravitreal anti-VEGF or steroid treatment for DMO in the past 4 months; PRP in the past 8 weeks and previous Iluvien therapy.

Key exclusion criteria that apply to systemic conditions include a glycated haemoglobin (HbA1c) level of more than 12%; blood pressure of more than 170/110 mm Hg; myocardial infarction, stroke, transient ischaemic attack, acute congestive cardiac failure or any acute coronary event within 6 months of randomisation; dialysis or renal transplant; pregnant or breastfeeding women or males and females who do not agree to use effective contraception during the study and for at least 3 months after the study has finished. Participation in an investigational trial involving an investigational medicinal product within 30 days of randomisation is also an exclusion.

### Randomisation

Randomisation will be via a bespoke web-based randomisation system hosted at the King's Clinical Trials Unit on a secure server. In total, 220 adult patients with PDR will be randomised 1:1 at the level of the individual using the method of minimisation incorporating a random element. The minimisation factors will be PDR status (treatment naïve vs active retinal NV post PRP), HbA1c (<8%, 8–10%, >10%), diastolic blood pressure (>90 vs ≤90 mm Hg), BCVA (54–69 vs ≥70 letters) and trial site.

## Trial interventions

### Intervention arm

Aflibercept (Bayer plc, Regeneron, Inc) is approved by the Food and Drug Administration and European Medicines Agency for wet age-related macular degeneration and macular oedema due to central retinal vein occlusion. Aflibercept will be provided by Bayer Healthcare Ltd. in accordance with its marketing authorisation. The Clinical Trials Manufacturing and Supplies Department, Pharmacy Production Department, Royal Free Hospital NHS Foundation Trust, will be responsible for packaging, labelling and Qualified Person (QP) releasing the drug prior to distribution to site. The physical, chemical and pharmaceutical properties, and formulation of aflibercept are provided in the current version of the SPC. The drug will be delivered in exactly the same dose and formulation as notified in the marketing authorisation for wet age-related macular degeneration and macular oedema due to central retinal vein occlusion.

All study eyes randomised to receive aflibercept will receive an intravitreal injection of aflibercept 2 mg/0.05 mL at baseline and at 4 and 8 weeks. Further treatment at week 12 is determined by the degree of regression of NV of disc and elsewhere on clinical examination with adequate visualisation of the entire retina and compared to the seven-field colour photographs or wide-field photography at screening. The patients will be categorised according to treatment response into three groups as shown in table 1: (1) no regression (2) partial regression and (3) total regression.

**Table 1:** Definition of regression patterns of retinal NV

Regression pattern	Definitions of regression patterns (compared to previous visit)
No regression	Any one or more of the following: No decrease in size or density of active NV Increase in area of active NV De novo active NV (flat or elevated) in an eye with pre-existing active NVs that have not regressed or partially regressed since the previous visit Iris or angle NV and NVG
Partial regression	Persistent active NV but a decrease in size or density of NV from the previous visit
Total regression	Any one or more of the following: Complete regression of NVE/D Regression of NV tissue to avascular fibrotic tissue Quiescent NV defined as inactive NV that, in the opinion of the investigator, does not require any further treatment
Reactivation	Reactivation can occur at any visit from week 16 and is defined as one or more of the following : Recurrence of NV De novo NV (flat or elevated) following at least 8 weeks of total regression

NV, neovascularisation; NVD, neovascularisation disc; NVE, neovascularisation elsewhere; NVG, neovascular glaucoma;.

From week 16, further treatment is determined by both regression and reactivation of NV on clinical examination with adequate visualisation of the entire retina and by comparing the four-field colour photographs or wide-field imaging done in the previous visit. The treatment response will be categorised into four groups: (1) no regression, (2) partial regression, (3) total regression and (4) reactivation as shown in table 1.

Further fields of colour retinal photographs or fluorescein fundus angiography may be performed at any visit if there is any doubt that a clinical feature represents retinal NV.

Aflibercept treatment may be deferred if an eye experienced an adverse event due to prior intravitreal injection, in cases of total vitreous haemorrhage with no clear view of the fundus, disease progression where risks of an injection outweigh the benefits or if the interval since the last aflibercept injection is <4 weeks.

### Comparator arm

PRP therapy, the current standard of care, will be the comparator and will be delivered as per routine clinical practice with emphasis on targeting retinal non-perfusion areas. In brief, treatment-naïve patients requiring PRP treatment will for the first time be initiated on it and completed in fractionated 2 weekly sessions up to and may include up to week 4 and then reviewed at week 12. Participants with persistent active new vessels that have had PRP previously and are randomised to the PRP arm will receive fill-in PRP in 1–2 two weekly sessions. From week 12, all patients in the PRP arm will be assessed for treatment response every 8 weeks and categorised exactly as the aflibercept arm.

PRP treatment can be done using any PRP delivery system including indirect PRP. If PRP has to be done as a day case, this should not be recorded as a serious adverse event (SAE) despite hospitalisation. PRP may be deferred if the media are too hazy to perform the procedure. In the ‘no regression category’ in both arms, PRP may be deferred if, in the opinion of the investigator, the eye has had adequate PRP and there is insufficient space for further fill-in PRP.

**Figure 2:**
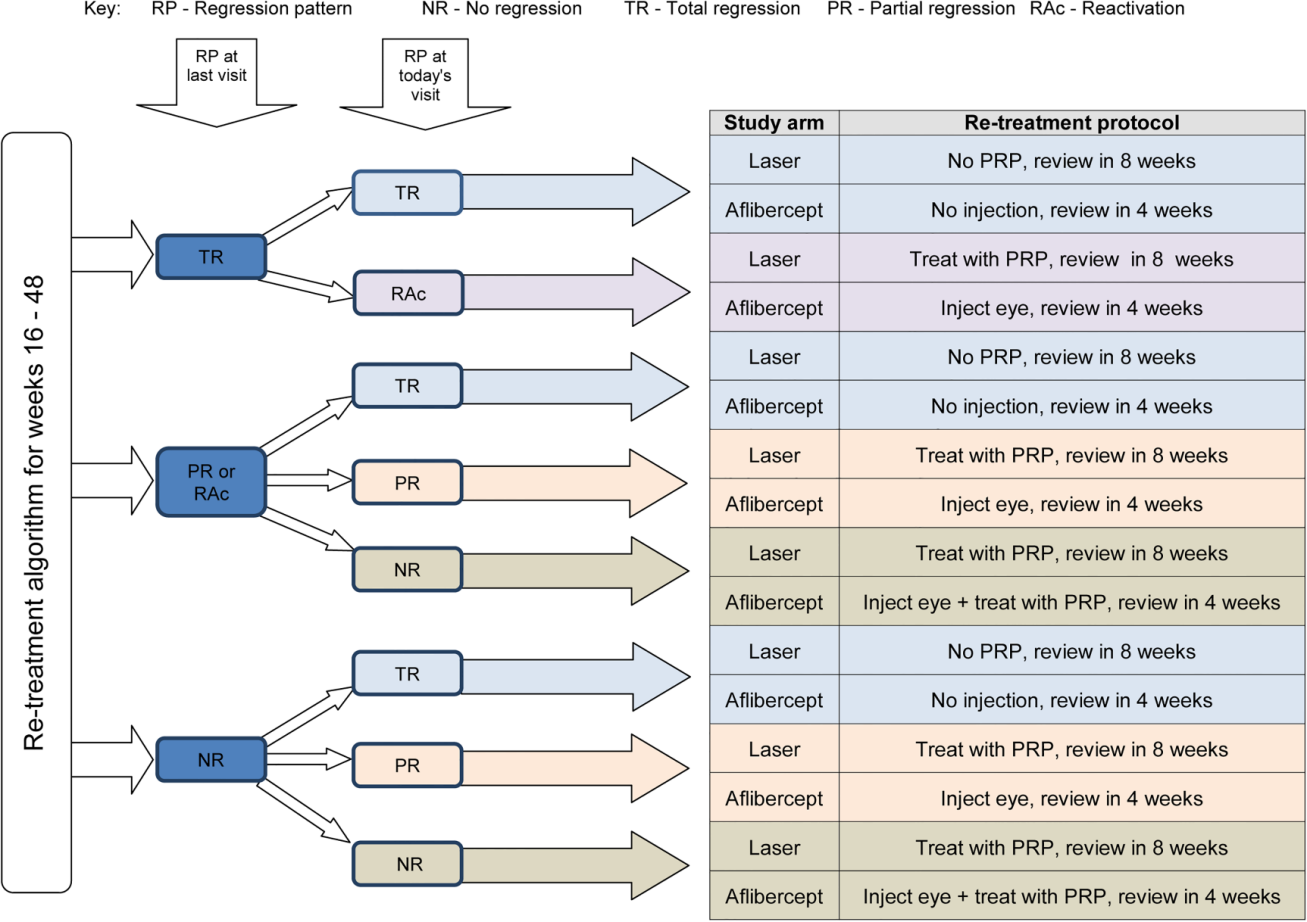
The regression patterns (RP) of the retinal neovascularisation is classified into the following: no regression (NR); total regression (TR); partial regression (PR) and reactivation (RAc) (PRP, panretinal photocoagulation).

Figure 2 shows the categorisation and treatment of patients from week 16.

### Masking

The research optometrists are the primary outcome assessors and will conduct the visual acuity tests at screening and at 12 and 52 weeks. The other tests of secondary outcome measures of visual fields and optical coherence tomography will also be performed by masked technicians. The participants will be advised at enrolment that they must not discuss the study arm they are in with these assessors. The retinal photographs at screening and at 12 and 52 weeks and fundus fluorescein angiography at screening and at 52 weeks will be graded by masked graders in the Independent Reading Centres within Network of Ophthalmic Reading Centres UK (NetwORC UK). These masking procedures will avoid both performance and detection bias. We will describe the completeness of outcome data for each outcome, including reasons for attrition and exclusions from the analysis.

## Sample size

The sample size calculation was performed using nQuery Advisor 4.0 software. The primary outcome is the change in BCVA measured as the ETDRS letter score from baseline to 52 weeks. On the basis of the objectives of this study and the potential deleterious effects on visual function by PRP, a non-inferiority margin of 5 L was judged to be clinically acceptable.^15–18^ In addition, this margin is less than the lower limit of the 95% CI for the comparison of immediate PRP with observation. This helps ensure that aflibercept is superior to observation alone in the event that it is found to be non-inferior to PRP. Therefore, in the wider patient population, if aflibercept is no more than five letters worse, then it will be defined to be non-inferior. The sample size is based on providing a 95% CI for the between-arm difference in mean change in visual acuity that will be sufficiently narrow to detect non-inferiority (by the CI lying entirely above the margin) with high power, while keeping a false declaration of non-inferiority to 5% through use of a statistical test applied at the two-sided 5% level of significance.

The SD of the change in visual acuity, after adjustment for baseline, is anticipated to be 10.3, based on the estimate from a relevant trial.^18^

With 110 patients randomised per arm (total 220), 182 will be followed up to the 52-week outcome (allowing for a 17% dropout or per protocol (PP) exclusion). This provides a 90% power to detect non-inferiority using a two-sided 95% CI from an analysis of covariance test with adjustment for baseline visual acuity.

## Outcomes

Primary outcome is the change in BCVA from screening to 52 weeks in the study eye measured in the ETDRS letter score at 4 m.

Secondary outcomes on visual functions are to measure the effect of intravitreal aflibercept therapy, relative to PRP on additional visual function and quality of life outcomes including change in BCVA at 12 weeks. These include:

1. Percentage of uniocular and binocular Esterman efficiency scores at 52 weeks;
2. Binocular visual acuity at 52 weeks;
3. Low luminance visual acuity at 52 weeks;
4. Visual acuity outcomes in terms of visual gain or loss;
5. Contrast sensitivity measured using the Pelli Robson chart at 52 weeks;
6. Change from baseline in vision-related quality of life measured using NEI-VFQ-25 and RetDQol at 52 weeks. NEI-VFQ 25 is a validated tool for vision-related quality of life.^19^ RetDQoL is a validated questionnaire specific for DR;^20^
7. Change from baseline in DR treatment satisfaction questionnaire (RetTSQ) scores at 52 weeks. RetTSQ is a DR treatment satisfaction questionnaire that has taken both anti-VEGF and PRP treatment into account when it was designed.^21^
8. Change from baseline in health-related quality of life at 52 weeks (EQ-5D, ICECAP-A and client service receipt inventory (CSRI)). The EQ-5D is a generic health-related quality of life measure, which will be collected at screening and at week 52 for health economics analysis.^22^ The ICECAP-A is a brief questionnaire which measures individual capability and well-being.^23^ A CSRI will be included to collect data on health and social care service use frequency and subsequent costs.

Anatomical outcomes include the regression patterns of new vessels at 12 weeks and the regression and reactivation patterns at 52 weeks. Additionally, the proportion of patients with 1-step and 3-step improvement or worsening of DR between treatment arms at 12 and 52 weeks will be reported.

Treatment-related outcomes include the proportions of naïve PDR and non-naïve PDR eyes that do not require PRP through 52 weeks after basic treatment of three loading doses of aflibercept in the aflibercept arm and after initial completion of PRP in the PRP arm.

The safety profile will be reported in terms of proportion of patients developing macular oedema, any de novo or increase in existing vitreous haemorrhage, new or increasing tractional retinal detachment, NVG and the requirement for vitrectomy for various indications.

### Cost-effectiveness analysis

From a public sector, multiagency perspective, the following economic analysis will be carried out:

1. Full costing of intravitreal aflibercept treatment and PRP, using national/local unit costs; we will collect information on patient service use using hospital data and a CSRI completed by patients.
2. A primary cost-effectiveness analysis, using PRP as the comparator. Change in BCVA will be used as the measure of effectiveness.
3. A secondary cost-utility analysis, using the EQ-5D as the measure of utility to generate a cost per QALY (ICER) and Cost Effectiveness Acceptability Curve (CEAC) for comparison with the National Institute for Health and Care Excellence (NICE) ceiling threshold of £20 000 to £30 000.^24^ ^25^

Sensitivity analyses will be conducted to see how key assumptions affect cost-effectiveness estimates.

### Mechanistic evaluation

The outcomes of the mechanistic evaluation will include regression of retinal NV at 12 and 52 weeks in terms of decimal disc area units; change in retinal vessel calibre and oxygen saturation and change in quantifiable areas of retinal non-perfusion. Independent grading of retinopathy and changes in retinal NV will be performed by graders in reading centres within the NetwORC UK.

## Data analysis plan

### Primary outcome analysis

Analyses will be on an intention-to-treat (ITT) basis. The primary outcome will be compared between arms primarily at the 52-week point and secondarily at the 12-week point using a linear mixed effects model with patient as a random effect to allow for within-patient correlation of repeated measures over time. The fixed effects will consist of the baseline of the outcome using the missing indicator method and any remaining minimisation stratifiers, including study site. These will be included as main effects and interactions with times. The test for non-inferiority will be one-sided at the 2.5% significance level, and presented as an estimated effect with a two-sided 95% CI compared with the non-inferiority margin.

For the analysis of the primary outcome, the mixed effects model will be refitted in a reduced PP population, excluding patients found to be ineligible at entry, and those not receiving the full randomised treatment up to and including the 8-week visit (whether due to discontinuation, exclusion or other reason for missing a randomised treatment in this period). Non-inferiority will only be concluded if this is declared by both the ITT analysis and the PP analysis at 52 weeks. Non-inferiority will also be assessed in ITT and PP populations at 12 weeks.

### Secondary outcome analysis

Secondary outcome analyses will be on an ITT basis only, and assessed with tests at the two-sided 5% level of significance. Continuous outcomes will be compared between arms using a linear mixed effects model, as specified for the primary outcome ITT analysis. Continuous and binary outcomes will be reported as differences in proportions assessed using χ^2^ tests. All tests will be two-sided at the 5% significance level and interpreted cautiously with a focus on interpreting effect sizes with 95% CIs. Safety outcomes will be reported as unadjusted patient proportions and rates within and between arms with 95% CIs using exact methods where appropriate.

### Sensitivity and other planned analyses

Sensitivity to the missing at random assumption made in the primary outcome analysis will be undertaken to assess sensitivity to the handling of the missing 52-week data, and to the use of concomitant treatments, and will be detailed in the statistical analysis plan.

If non-inferiority is concluded, superiority will be assessed, and also the effect on the primary outcome will be presented with 95% CI within baseline retinopathy status subgroups: naïve PDR and non-naïve PDR.

## Ethical issues

The main ethical issues in relation to this study are the use of intravitreal injections. However, this is now the standard of care for wet age-related macular degeneration, DMO and retinal vein occlusion. There are at least five extra visits that the participants need to undergo in excess of the standard of care. The precise risks and benefits of participating in the clinical study will be outlined in patient information sheets, formulated with service user involvement.

Participants in the mechanistic substudy have to undergo retinal oximetry, an additional non-invasive imaging of the retina at baseline, as well as at 12 and 52 weeks and at the point of withdrawal. There are no known risks for retinal oximetry.

Participants will be treated with the standard of care (PRP) if the disease recurs after they have completed the study. This information is reflected in the patient information sheet.

Any breach of confidentiality will be minimised by adherence to the UK Data Protection Act 1998 and the approved protocol. **T**he trial will be employing an electronic data capture system (Infermed MACRO). Access to the system will be restricted to authorised site personnel.

Aflibercept will be delivered in exactly the same dose and formulation as notified in the marketing authorisation for wet age-related macular degeneration and macular oedema due to central retinal vein occlusion. All adverse events will be recorded in the electronic case report form (eCRF) throughout the study regardless of their severity or relation to study participation.

The protocol is approved by the National Research Ethics Service Committee London—South East (14/LO/0203). The chief investigator will submit an annual report of all SAEs to the Sponsor, and the Research Ethics Committee and the MHRA. The Data Monitoring and Ethics Committee (DMEC) will be provided listings of all SAEs on an ongoing basis.

The study may be prematurely discontinued on the basis of new safety information, or for other reasons given by the DMEC and/or Trial Steering Committee (TSC), Sponsor or Research Ethics Committee concerned.

## Dissemination plan

The research will be published in high-impact ophthalmology and diabetes journals and presented in scientific meetings in the retinal section in ophthalmology. We also plan to present in key conferences on diabetes. Each participant will receive a summary of the results and discussion. The study results will also be disseminated to the Diabetes Research Network and Diabetes UK so that service users and health professionals will be informed of the results. All stakeholders including health policymakers will have access to the findings of this research.

## References

[1] Abbate M, Cravedi P, Iliev I Prevention and treatment of diabetic retinopathy: evidence from clinical trials and perspectives. Curr Diabetes Rev 2011;7:190–200. 10.2174/157339911795843168 21438851

[2] [No authors listed] Early photocoagulation for diabetic retinopathy. ETDRS report number 9. Early Treatment Diabetic Retinopathy Study Research Group. Ophthalmology 1991;98(5 Suppl):766–85. 10.1016/S0161-6420(13)38011-7 2062512

[3] [No authors listed] Preliminary report on effects of photocoagulation therapy. The Diabetic Retinopathy Study Research Group. Am J Ophthalmol 1976;81:383–96. 10.1016/0002-9394(76)90292-0 944535

[4] Stefánsson E Ocular oxygenation and the treatment of diabetic retinopathy. Surv Ophthalmol 2006;51:364–80. 10.1016/j.survophthal.2006.04.005 16818083

[5] Aiello LM Perspectives on diabetic retinopathy. Am J Ophthalmol 2003;136:122–35. 10.1016/S0002-9394(03)00219-8 12834680

[6] Bailey CC, Sparrow JM, Grey RH The National Diabetic Retinopathy Laser Treatment Audit. III. Clinical outcomes. Eye (Lond) 1999;13:151–9. 10.1038/eye.1999.42 10450373

[7] Shima DT, Adamis AP, Ferrara N Hypoxic induction of endothelial cell growth factors in retinal cells: identification and characterization of vascular endothelial growth factor (VEGF) as the mitogen. Mol Med 1995;1:182–93. 8529097 PMC2229943

[8] Lange CA, Stavrakas P, Luhmann UF Intraocular oxygen distribution in advanced proliferative diabetic retinopathy. Am J Ophthalmol 2011;152:406–12. 10.1016/j.ajo.2011.02.014 21723532

[9] Miller JW, Adamis AP, Shima DT Vascular endothelial growth factor/vascular permeability factor is temporally and spatially correlated with ocular angiogenesis in a primate model. Am J Pathol 1994;145:574–84. 7521577 PMC1890317

[10] Aiello LP, Avery RL, Arrigg PG Vascular endothelial growth factor in ocular fluid of patients with diabetic retinopathy and other retinal disorders. N Engl J Med 1994;331:1480–7. 10.1056/NEJM199412013312203 7526212

[11] Adamis AP, Shima DT, Tolentino MJ Inhibition of vascular endothelial growth factor prevents retinal ischemia-associated iris neovascularization in a nonhuman primate. Arch Ophthalmol 1996;114:66–71. 10.1001/archopht.1996.01100130062010 8540853

[12] Aiello LP, Pierce EA, Foley ED Suppression of retinal neovascularization in vivo by inhibition of vascular endothelial growth factor (VEGF) using soluble VEGF-receptor chimeric proteins. Proc Natl Acad Sci USA 1995;92:10457–61. 10.1073/pnas.92.23.10457 7479819 PMC40630

[13] Ip MS, Domalpally A, Hopkins JJ Long-term effects of ranibizumab on diabetic retinopathy severity and progression. Arch Ophthalmol 2012;130:1145–52. 10.1001/archophthalmol.2012.1043 22965590

[14] Salam A, D'Costa J, Sivaprasad S Treatment of proliferative diabetic retinopathy with anti-VEGF agents. Acta Ophthalmol 2011;89:405–11. 10.1111/j.1755-3768.2010.02079.x 21294854

[15] Martin DF, Maguire MG, Fine SL, et al., Comparison of Age-related Macular Degeneration Treatments Trials (CATT) Research Group. Ranibizumab and bevacizumab for treatment of neovascular age-related macular degeneration: two-year results, Ophthalmology 2012;119:1388–98. 10.1016/j.ophtha.2012.03.053 22555112 PMC3389193

[16] Rosser DA, Cousens SN, Murdoch IE How sensitive to clinical change are ETDRS logMAR visual acuity measurements? Invest Ophthalmol Vis Sci 2003;44:3278–81. 10.1167/iovs.02-1100 12882770

[17] Chakravarthy U, Harding SP, Rogers CA, et al., IVAN Study Investigators. Ranibizumab versus bevacizumab to treat neovascular age-related macular degeneration: one-year findings from the IVAN randomized trial. Ophthalmology 2012;119:1399–411. Erratum in: *Ophthalmology* 2012 Aug;119(8):1508. *Ophthalmology* 2013;120(9):1719 10.1016/j.ophtha.2012.04.015 22578446

[18] González VH, Giuliari GP, Banda RM Intravitreal injection of pegaptanib sodium for proliferative diabetic retinopathy. Br J Ophthalmol 2009;93:1474–8. 10.1136/bjo.2008.155663 19692371

[19] Mangione CM, Lee PP, Gutierrez PR, et al., National Eye Institute Visual Function Questionnaire Field Test Investigators. Development of the 25-item National Eye Institute Visual Function Questionnaire. Arch Ophthalmol 2001;119:1050–8. 10.1001/archopht.119.7.1050 11448327

[20] Brose LS, Bradley C Psychometric development of the individualized Retinopathy-Dependent Quality of Life Questionnaire (RetDQoL). Value Health 2010;13:119–27. 10.1111/j.1524-4733.2009.00589.x 19695003

[21] Brose LS, Bradley C Psychometric development of the retinopathy treatment satisfaction questionnaire (RetTSQ). Psychol Health Med 2009;14:740–54. 10.1080/13548500903431485 20183546

[22] Janssen MF, Birnie E, Bonsel GJ Quantification of the level descriptors for the standard EQ-5D three-level system and a five-level version according to two methods. Qual Life Res 2008;17:463–73. 10.1007/s11136-008-9318-5 18320352 PMC2275305

[23] Al-Janabi H, Peters TJ, Brazier J An investigation of the construct validity of the ICECAP-A capability measure. Qual Life Res 2013;22:1831–40. 10.1007/s11136-012-0293-5 23086535 PMC3764327

[24] Council MR. A framework for development and evaluation of RCT. BMJ 2009;339:b3496 10.1136/bmj.b3496 19744976 PMC2741564

[25] Edwards RT, Ceilleachair A, Bywater T Parenting programme for parents of children at risk of developing conduct disorder: cost effectiveness analysis. BMJ 2007;334:682–5. 10.1136/bmj.b3496 17350965 PMC1839236

